# Longitudinal relationships of psychotic-like experiences with suicidal ideation and self-harm in adolescents

**DOI:** 10.1007/s00787-023-02299-1

**Published:** 2023-09-23

**Authors:** Rui Zhou, Jerome Clifford Foo, Asuka Nishida, Sayoko Ogawa, Fumiharu Togo, Tsukasa Sasaki

**Affiliations:** 1 Department of Physical and Health Education, Graduate School of Education, The University of Tokyo, 7-3-1 Hongo, Bunkyo-Ku, Tokyo, 113-0033 Japan; 2 Institute for Psychopharmacology, Medical Faculty Mannheim, Central Institute of Mental Health, University of Heidelberg, Mannheim, Germany; 3 Department of Genetic Epidemiology in Psychiatry, Medical Faculty Mannheim, Central Institute of Mental Health, University of Heidelberg, Mannheim, Germany; 4 Department of Psychiatry, College of Health Sciences, University of Alberta, Edmonton, Canada

**Keywords:** Psychotic experiences, Suicidal ideation, Self-harm, Psychological distress, Adolescents, Mental health

## Abstract

Research in adolescents suggests associations between psychotic-like experiences (PLEs) and self-injurious thoughts and behaviors (SITBs), but insights into their temporal relationship, which may inform prediction, have been limited. Psychological distress (PD; symptoms of depression and anxiety) has been related to both PLEs and SITBs, and may modulate this relationship. Given that PLEs have been linked to the development of several mental disorders, and the relationships between SITBs and suicide, it is important to better understand their relationship. The present study sought to investigate these factors using a longitudinal school-based design. Adolescents (*n* = 1685, ages 12–18) completed annual self-report assessments (6 time points) on PLEs, SITBs (suicidal ideation (SI) and self-harm (SH)), as well as PD. The longitudinal associations between PLEs and SITBs were analyzed, employing two cross-lagged panel models (CLPMs), with and without adjustment for PD. Unadjusted CLPMs revealed significant bidirectional temporal associations between PLEs and SITBs (both SI and SH), suggesting that PLEs both predicted and were predicted by SITBs. When adjusting for PD, the effect of SI on PLEs remained significant, but not PLEs on SI; bidirectional associations between PLEs and SH also remained significant. A bidirectional longitudinal relationship where both PLEs and SITBs can precede (and perhaps predict) each other was suggested in adolescents. PD may play a particular role in situations where PLEs are followed by SI. Heightened awareness about relationships between these phenotypes may be an important step toward facilitating timely interventions for both mental disorders and suicide.

## Introduction

Suicide has been the second leading cause of death in youth and young adults over the past decade [1]. Self-injurious thoughts and behaviors (SITBs), such as suicidal ideation (SI) and self-harm (SH) are the main predictors of future suicide [2–4]. SITBs have the highest prevalence during adolescence [3], and are associated with a range of negative outcomes later in life, including suicide mortality, ill-health, low educational attainment, and unemployment [2, 3, 5].

Psychotic-like experiences (PLEs), which refer to sub-threshold psychotic symptoms such as subclinical hallucinatory and delusional experiences [6], have been consistently shown to be associated with the development of several mental disorders [7–9], as well as SITBs [10, 11], particularly in adolescents [12–16]. A cross-national analysis of over 30,000 subjects (from 19 countries) has suggested that children and adolescents who have experienced PLEs had 2–3-fold increased odds of subsequent suicidal thoughts and behaviors [12].

Although PLEs have been considered a risk factor for SITBs [10, 11], a recent study has proposed a suicidal drive hypothesis, which suggests that SITBs may prompt the emergence of PLEs in some individuals, as a means of externalizing the self-directed threat that arises from SITBs [17]. Three longitudinal studies, utilizing two or three waves of data (collected over 2, 4, 6 years, respectively) have investigated this hypothesis, yielding inconsistent findings: while two studies supported a temporal association between self-injurious/suicidal behaviors and PLEs/auditory hallucinations [18, 19], the other study found no such temporal link [20]. These disparate results may stem from differences in assessments, analytical techniques, and study design/length, as well as confounding factors that have been linked to both phenomena [21].

Psychological distress (PD; symptoms of depression and anxiety) is a well-established risk factor for SITBs [22–24], and has also been linked with PLEs in adolescents in several studies [25–27]. Adolescents suffering from both PLEs and depression are at significantly higher risk of SITBs than those with depression only [24, 28, 29]. However, the effect size of PLEs on SITBs is smaller when adjusted for depression [10], suggesting that PD may play a role in the relationship between PLEs and SITBs. Additionally, a longitudinal population study indicated that PLEs increase the risk of SITBs beyond what is explained by co-existing psychopathology (e.g., mental disorders) [11]. Therefore, further research is necessary to understand the impact of PD on the relationship between PLEs and SITBs.

Given the unclear results in prior studies, to achieve a better understanding of (temporal) relationships between PLEs and SITBs, research looking at multiple time points with shorter lags, as well as accounting for PD, is needed. This research is especially needed in adolescence, as this developmental stage is characterized by significant changes in brain structure and function [30, 31], the heightened susceptibility to psychopathological conditions like psychosis [32, 33], and the critical emergence of suicidality and self-harm [34, 35]. Therefore, when investigating the longitudinal relationship between PLEs and SITBs in adolescents, it is important to carefully consider the duration of the intervals being studied. A related factor which needs investigation is gender as development of boys and girls occurs on different time scales. Some studies have shown that PLEs and SITBs are more common in girls than in boys [36–41]. However, to date, no studies have investigated whether the associations between PLEs and SITBs differ in boys and girls, which may be relevant to the development of intervention strategies.

The purpose of this study was to investigate the potential bidirectional predictive relationship between PLEs and SITBs in adolescents. We analyzed up to 6 years of annual survey data for each individual to identify any longitudinal patterns in these behaviors in the whole sample, boys and girls. Our analysis was conducted using cross-lagged panel models (CLPMs), which enabled us to examine multiple time points simultaneously and determine the direction and strength of any associations between PLEs and SITBs.

## Methods

### Participants

The data were obtained from a longitudinal survey of adolescent mental health status conducted from 2011 to 2019 in a combined junior and senior high school (grades 7–12) in Tokyo, Japan. The survey was conducted annually, and the participants in the survey ranged from 699 to 707 (participation rate: 97.7–99.0%) each year, with a total of 1685 students (ages 12–18) participating through the course of the study (836 boys and 844 girls, 5 not indicated). After excluding the 5 individuals without gender information, data of 1680 participants were included in the final analysis.

### Procedure

The study was approved by the Ethics Committee of the Life Science Committee of the University of Tokyo (#15–128) and by the Research Department of the school. Consent was obtained from school principals, parents, and students. Students and their parents were informed of the aims and contents of the survey via written documents before the surveys. On the day of the survey, teachers explained that any students who did not wish to participate could leave the classroom or turn in a blank questionnaire. Students completed questionnaires and sealed them in provided envelopes, indicating their student IDs. IDs were transferred to researchers; answers were studied anonymously but longitudinal follow-up was possible.

### Measures

#### Psychotic-like experiences (PLEs)

PLEs occurring in the past 6 months were assessed using five items from the Schizophrenia section of the Japanese version of the Diagnostic Interview Schedule for Children (DISC-C) [42]. Two items assessed for hallucinatory experiences (auditory and visual) and three items assessed delusional experiences: (1) thoughts being read, (2) feeling spied upon, and (3) receiving special messages. The DISC-C has been widely used to assess PLEs in adolescents in previous studies [13, 16, 25, 43, 44]. All answers were given on a four-point scale: ‘no’, ‘maybe’, ‘yes, once’, and ‘yes, twice or more’. We defined students who answered ‘yes, once’ or ‘yes, twice or more’ on any of the five items as having experienced PLEs in the survey year.

#### SITBs (suicidal ideation and self-harm)

SITBs were assessed by two questions. SI was measured using the following question: “Do you currently have thoughts that life is no longer worth living?” [45]. We opted for this phrasing to ensure inclusivity and capture a wide range of ideation experiences, from contemplation to explicit wishes. The participants selected one of four responses: “No”, “Probably Not”, “Possibly Yes” and “Yes”. Students who answered “Yes” or “Possibly Yes” were defined as having suicidal ideation. SH was assessed by the question: “Did you intentionally injure yourself within the past year?” (‘yes’/‘no’), and when the answer was “yes”, the participants were defined as having self-harmed.

#### Psychological distress (PD)

PD was evaluated using the Japanese version of the 12-item General Health Questionnaire (GHQ-12) [46]. The GHQ-12 is a widely used self-reported screening instrument which assesses perceived psychological wellbeing over the past month in the general population. This assessment uses a four-point Likert scale (0-1-2-3) with binary item scoring, where two responses (0: Not at all, 1: Same as usual) are coded as 0, and two responses (2: Rather more than usual, 3: Much more than usual) are coded as 1 [0-0-1-1]). Individual item scores were summed to form a total score ranging from 0 (best possible score) to 12 (worst possible score).

### Statistical analysis

Descriptive statistics including the prevalence of PLEs, SI and SH, and average scores of GHQ-12 across grades were calculated. Pearson’s Chi-squared tests were used to analyze whether there were gender differences in PLEs and SITBs in grade level. T-tests were used to compare PD in individuals with and without PLEs and SITBs.

CLPMs were used to examine longitudinal relationships between PLEs and SITBs. CLPMs are commonly used in longitudinal research to clarify the temporal precedence and directionality of associations between variables, because they estimate the cross-lagged effects of one variable on the other and vice versa simultaneously considering multiple time points [47]. The CLPM analyses were conducted in Mplus version 8.3, using the MLR (Maximum Likelihood Robust) estimator, which is designed to handle situations where residuals may not be normally distributed and when there is non-independence of observations [48]. Missing data were handled using Full Information Maximum Likelihood (FIML).

**Fig. 1:**
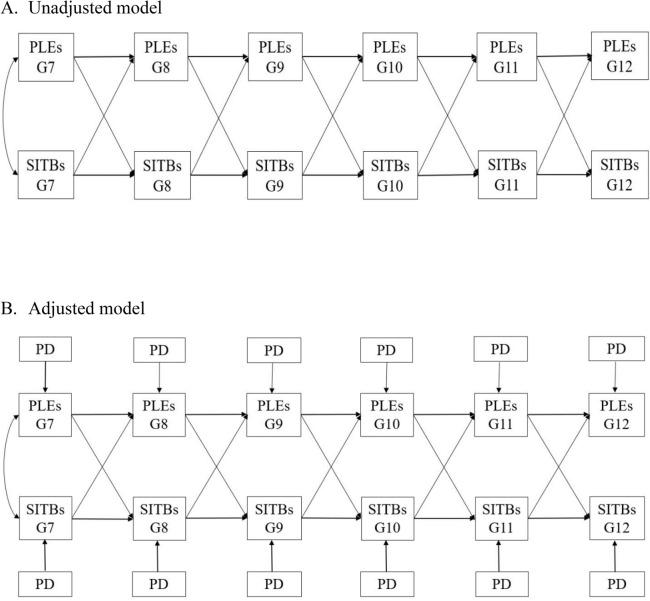
Path diagrams of the longitudinal relationship between PLEs and SITBs. *PLEs* Psychotic-like experiences, *SITBs* Self-injurious thoughts and behaviors (suicidal ideation/self-harm), *PD* Psychological distress, *G* Grade

In this study, we assumed that the longitudinal associations between variables were constant across time points, and therefore, autoregressive and cross-lagged effects were constrained equally (i.e., the effect of PLEs at T1 on PLEs/SITBs at T2 was equal to the effect of PLEs at T2 on PLEs/SITBs at T3, and so on). We specified 2 CLPMs to examine the longitudinal relationship between PLEs and SITBs. The first included the paths between the two distinct variables across adjacent time points (i.e., cross-lagged paths) and the five adjacent measurements of PLEs and SITBs (i.e., autoregressive paths) (unadjusted model, Fig. 1A**)**; the second model added PD as a covariate to control for its confounding effect on the associations (adjusted model, Fig. 1B). These two models were specified for both PLEs with SI and PLEs with SH. In addition, all analyses were also performed separately by gender.

## Results

### Descriptive statistics

Table 1 displays the prevalence rates of PLEs, SITBs, and the average GHQ-12 score in adolescents (ages 12–18, grades 7–12). The prevalence rates of PLEs, SITBs, and PD were significantly higher in girls than boys across all grades. Individuals with PLEs and SITBs exhibited significantly higher levels of PD compared to those without these experiences (all *p* < 0.001, not shown in the table). The prevalence of PLEs significantly decreased, while SI and PD increased significantly with increasing grade (all *p* < 0.001). No significant differences were found in SH across grades.

**Table 1:** Prevalence rates (%) of PLEs, SI and SH, average PD score (GHQ-12)

	Whole sample (*N* = 1680)				Boys (*N* = 836)				Girls (*N* = 844)			
	PLEs	SI	SH	PD	PLEs	SI	SH	PD	PLEs	SI	SH	PD
Grade 7	10.8	3.5	6.6	1.7	9.1	3.4	5.6	1.5	12.5	3.6	7.5	2.0
Grade 8	10.3	6.5	7.3	2.3	9.1	5.1	6.2	2.0	11.5	7.9	8.5	2.7
Grade 9	6.5	6.9	6.1	2.4	4.6	5.2	5.8	1.9	8.7	8.5	6.3	2.9
Grade 10	6.5	7.6	6.1	2.7	5.3	6.7	5.8	2.1	7.7	8.4	6.5	3.3
Grade 11	7.0	8.5	5.3	3.1	5.2	6.7	3.9	2.6	8.7	10.3	6.7	3.6
Grade 12	6.6	10.1	6.5	3.5	5.2	10.4	4.4	2.8	8.1	9.9	8.7	4.1

Grades 7–12 correspond to ages 12–18

*PLEs* Psychotic-like experiences, *SI* Suicidal ideation, *SH* Self-harm, *PD* Psychological distress

### Longitudinal relationships between PLEs and SITBs

#### CLPMs analysis of PLEs and SI

Table 2 presents the results of CLPMs for the longitudinal relationships between PLEs and SITBs. In the unadjusted model, the cross-lagged path estimates between PLEs and SI were significant in both directions in the whole sample (PLEs → SI: odds ratio (OR) = 1.98, 95% CI 1.33–2.95; SI → PLEs: OR = 2.67, 95% CI 1.85–3.85). After adjusting for PD, the cross-lagged effects were attenuated and only the effect of SI on PLEs remained significant (OR = 1.58, 95% CI 1.04–2.38).

**Table 2:** Summary of CLPM parameters

Cross-lagged path	Sample	Unadjusted OR (95% CI)	Adjusted OR (95% CI)^a^
PLEs → SI	Total	**1.98 (1.33–2.95)**	1.08 (0.70–1.68)
	Boys	1.33 (0.65–2.72)	0.54 (0.23–1.29)
	Girls	**2.36 (1.44–3.85)**	1.63 (0.96–2.78)
SI → PLEs	Total	**2.67 (1.85–3.85)**	**1.58 (1.04–2.38)**
	Boys	**3.04 (1.70–5.42)**	**2.09 (1.10–3.99)**
	Girls	**2.32 (1.43–3.77)**	1.26 (0.74–2.16)
PLEs → SH	Total	**3.20 (2.27–4.52)**	**2.30 (1.60–3.32)**
	Boys	**2.23 (1.18–4.24)**	1.52 (0.76–3.05)
	Girls	**3.63 (2.37–5.57)**	**2.80 (1.77–4.43)**
SH → PLEs	Total	**2.39 (1.68–3.39)**	**1.74 (1.21–2.49)**
	Boys	1.56 (0.77–3.19)	1.27 (0.63–2.58)
	Girls	**2.79 (1.85–4.21)**	**1.92 (1.23–3.00)**

Bold indicates significant odds ratio (*p* < 0.05)

*SI* Suicidal ideation, *SH* Self-harm, *OR* Odds ratio, *95% CI* 95% Confidence interval

^a^Adjusted OR: Adjusted for psychological distress

When stratified by gender, in the unadjusted model, the effect of SI on PLEs was significant for both boys and girls (boys: OR = 3.04, 95% CI 1.70–5.42; girls: OR = 2.32, 95% CI 1.43–3.77), while the effect of PLEs on SI was significant only in girls (OR = 2.36, 95% CI 1.44–3.85). After adjusting for PD, the effect of SI on PLEs remained significant in boys (OR = 2.09, 95% CI 1.10–3.99); the estimates in both directions were no longer significant in girls.

#### CLPMs analysis of PLEs and SH

As shown in Table 2, in the unadjusted model, both the effect of PLEs on SH and the effect in the opposite direction were significant in the whole sample (OR = 3.20, 95% CI 2.27–4.52 and OR = 2.39, 95% CI 1.68–3.39; respectively). Following PD adjustment, the effects in both directions were attenuated but remained significant (PLEs → SH: OR = 2.30, 95% CI 1.60–3.32; SH → PLEs: OR = 1.74, 95% CI 1.21–2.49).

When stratified by gender, girls showed significant associations between PLEs and SH in both directions regardless of adjustment (Unadjusted model: PLEs → SH, OR = 3.63, 95% CI 2.37–5.57; SH → PLEs: OR = 2.79, 95% CI 1.85–4.21; Adjusted model: PLEs → SH: OR = 2.80, 95% CI 1.77–4.43; SH → PLEs: OR = 1.92, 95% CI 1.23–3.00), while boys only showed a significant association between PLEs and subsequent SH in the unadjusted model (OR = 2.23, 95% CI 1.18–4.24).

## Discussion

The present study sought to elucidate the temporal direction of association between PLEs and SITBs in adolescents, employing longitudinal data from multiple time points. Notably, our findings suggest that PLEs exhibit bidirectional relationships with both SI and SH, with PD potentially acting as shared risk factor for the association between PLEs and SI, but not SH. These insights contribute to our understanding of the complex interplay between PLEs and SITBs, and highlight the importance of considering multiple factors in the assessment and management of these conditions for suicide prevention.

Our study provides evidence of a bidirectional association between PLEs and SI in adolescents. Our findings reveal that PLEs are associated with subsequent SI and vice versa, suggesting a reciprocal longitudinal association between the two. However, after adjusting for non-psychotic psychopathology, specifically PD, the association was significantly attenuated and only the effect of SI on PLEs remained significant in the whole sample. These results may suggest that PD could be a shared risk factor for both PLEs and SI. This is in line with previous research, which also suggests that PD may complicate the association between PLEs and SI [10, 21, 49]. In addition, both PLEs and SI are proposed to be trans-diagnostic indicators of severe psychopathology, which frequently co-occur in the context of severe social stressors [49, 50]. Overall, these findings suggest that PLEs and psychological factors could be considered together when assessing suicide risk in adolescents.

Self-harm has been associated with PLEs in several previous studies; however, only a limited number of longitudinal studies have focused on this association in adolescents, suggesting PLEs can be a predictor of SH [24, 51]. Our multi-timepoint longitudinal study on adolescents found a bidirectional relationship between PLEs and SH, with each factor preceding the other. Specifically, we observed that adolescents with PLEs were three times more likely to exhibit self-harm behaviors in the following year, while those with self-harm behaviors had a doubled risk of experiencing PLEs in the subsequent year. The observed impact of PLEs on SH in our study aligns with a systematic review and meta-analysis indicating a pooled OR of 3.20 (95% CI 2.33–4.40) for this association [10]. Even after adjusting for PD, the bidirectional relationship remained significant, indicating that PLEs and SH could be predictive of each other robustly.

Furthermore, our study found that while PLEs are associated with both SI and SH in adolescents, co-occurring PD may have a more significant effect on the PLE-SI relationship than the PLE-SH relationship. This result may reflect the different roles that PD plays in the mechanism of PLEs with SI and SH. Specifically, SI might be more closely linked to emotional states, such as depression and anxiety, which can amplify the experience of PLEs and increase likelihood of suicidal thoughts [52]. In contrast, SH may be more closely linked to other factors such as impulsivity or low self-esteem [37, 53].

Psychological distress may mediate the effects of PLEs on suicidal ideation. Research in adolescents indicates that PLEs might only be associated with concurrent and future suicide attempts when they are accompanied by general PD [24], and in college students, distress stemming from PLEs was able to predict a heightened risk of SI [50]. A recent study in children suggests that distress inherent to PLEs is both a mediator and moderator of the relationship between PLEs and suicidal ideation and behavior [54]. Our findings, taken together with the literature, appear to suggest that it is not necessarily the PLEs per se but the distress caused by them which may lead to SITBs.

Our results suggest that the longitudinal relationships between PLEs and SITBs differ between adolescent boys and girls. Our study found that girls have a higher prevalence of PLEs and SITBs compared to boys, which is consistent with prior research [40, 41, 55]. Moreover, after adjusting for PD, the bidirectional relationship between PLEs and SH remained significant only in girls, suggesting that the effects of PD on the relationships could be gender specific. Our results also suggest that while the bidirectional relationship of PLEs-SH is more robust in girls, the effect of SI on PLEs is only significant in boys. The finding may be in line with prior evidence that girls are more likely to engage in non-suicidal self-injury, whereas boys are more likely to exhibit aggressive forms of SITBs [34]. The differences between boys and girls in the symptoms and risk factors of PLEs and SITBs have important implications for diagnosis and treatment, as highlighted in studies of other psychiatric disorders [56]. Our findings support the need for further investigations into the gender-specific relationship between PLEs and SITBs to better inform prevention and intervention strategies. Furthermore, it is worth noting that the gender differences observed in our study may also stem in part from the lower prevalence of SITBs in boys, potentially impacting the statistical power to detect effects.

It is crucial to identify and address the underlying risk factors that lead to suicidal behaviors among adolescents. Our study suggests that screening for PLEs and PD in schools may help identify students at high risk for SITBs and inform tailored interventions to prevent future harm. Parents, school staff, and clinicians should remain vigilant and provide support to adolescents exhibiting PLEs, as these may be early indications of PD and an increased risk of future self-harm. While SITBs are generally better predictors of future suicide and should be screened for, there are also situations where individuals might be hesitant to disclose or report them accurately, for reasons such as stigma or difficulty in articulating their experiences. Adolescents, in particular, may be more likely to respond to questions about PLEs than SITBs [57]. Our findings suggest the potential of using screening for PLEs and PD as a possible step in the prevention of adolescent suicide. In addition, we note that adolescents at clinical/ultra-high risk (CHR/UHR) of psychosis also report PLEs [58]. The prevalence of SITBs in these groups may be similar to that of in people with diagnosed psychotic disorders [59], further suggesting the need for careful monitoring of people experiencing PLEs.

## Limitations

This study had several limitations. First, study variables were self-reported and could be more prone to measurement error and recall bias than interviews. Second, the study sample was from a single school in Tokyo, thus caution is needed when generalizing to other populations. Third, the assessment of SITBs was based on brief questions. Future studies may benefit from the use of more detailed and nuanced questionnaires to evaluate SITBs along the spectrum of suicide risk, including the intensity of suicidal thoughts and the presence of specific plans. Additionally, the assessment of PLEs in this study did not capture the full range of psychotic symptoms, including negative symptoms. Also, while we have employed a widely used screening method, the small number of self-report items in the assessment may leave some room for misinterpretation by participants, picking up on normative rather than psychotic-like experiences. Fourth, this study did not adjust for other potential confounding factors, such as traumatic life events (e.g., bullying, physical or sexual abuse/assault) and low self-esteem [51]. Future research should consider including these potential confounders to provide a more comprehensive understanding of the relationship between PLEs and SITBs. Finally, it is noteworthy that five participants did not provide gender information, possibly due to the fact that non-binary categories were not integrated in the assessment (which began in 2011, when awareness of gender identity related issues was less than at present). This underscores the need to embrace more comprehensive and up-to-date methodologies for gender representation in forthcoming research.

## Conclusion

This study has shed light on the bidirectional longitudinal relationship between PLEs and SITBs in adolescents and has shown that PLEs are associated with subsequent SITBs, and SITBs also associated with subsequent PLEs. The relationship between PLEs and suicidal ideation may be partly explained by psychological distress. Heightened awareness about the relationships between these phenotypes may help in the assessment of risk for the development of both mental disorders and suicide, and be an important step toward facilitating timely interventions.

## Data Availability

The datasets generated during and/or analyzed during the current study are available from the corresponding author upon reasonable request.
